# Investigations on the structural and optoelectronic characteristics of cadmium-substituted zinc selenide semiconductors

**DOI:** 10.3389/fchem.2023.1299013

**Published:** 2023-12-15

**Authors:** Muhammad Aamir Iqbal, Sunila Bakhsh, Mujtaba Ikram, Muhammad Sohail, Md. Rasidul Islam, Salim Manoharadas, Jeong Ryeol Choi

**Affiliations:** ^1^ School of Materials Science and Engineering, Zhejiang University, Hangzhou, China; ^2^ Department of Physics, Balochistan University of Information Technology, Engineering and Management Sciences, Quetta, Pakistan; ^3^ Institute of Chemical Engineering and Technology (ICET), University of Punjab, Lahore, Pakistan; ^4^ Department of Physics, University of Balochistan, Quetta, Pakistan; ^5^ Department of Electrical and Electronic Engineering, Bangamata Sheikh Fojilatunnesa Mujib Science and Technology University, Jamalpur, Bangladesh; ^6^ Department of Botany and Microbiology, College of Science, King Saud University, Riyadh, Saudi Arabia; ^7^ School of Electronic Engineering, Kyonggi University, Suwon, Gyeonggi–do, Republic of Korea

**Keywords:** Zn_1-x_Cd_x_Se semiconductors, cubic symmetry, density functional theory, concentration dependency, density of states, bandgap, optical properties

## Abstract

A change in the composition and dopant content of selective atoms in a material leads to their new desired properties by altering the structure, which can significantly improve the performance of relevant devices. By acknowledging this, we focused on characterizing the optoelectronic and structural properties of cadmium-substituted zinc selenide (Zn_1-x_Cd_x_Se; 0 ≤ X ≤ 1) semiconductors using density functional theory (DFT) within the generalized gradient approximation (GGA), EV-GGA, and mBJ approximations. The results proved the cubic symmetry of the investigated materials at all Cd concentrations (0, 0.25, 0.50, 0.75, and 1). Although a linear surge in the lattice constant is observed with the change in Cd content, the bulk modulus exhibits a reverse trend. These materials are observed to be direct bandgap semiconductors at all Cd concentrations, with a decrease in electronic bandgap from 2.76 eV to 1.87 eV, and have isotropic optical properties, showing their potential applicability as a blue-to-red display. The fundamental optical properties of the materials, such as optical conductivity, reflectance, refractive index, absorption, and extinction coefficient, are also discussed. These outcomes provide a computational understanding of the diverse applications of Zn_1-x_Cd_x_Se semiconductors in optoelectronic, photonic, and photovoltaic devices, particularly for a visible-range display.

## 1 Introduction

Technological progress in semiconductor engineering has been proven to draw extraordinary advancements in a variety of industrial applications by opening a plethora of innovative devices with unmatched outcomes. For instance, direct bandgap semiconductors have been widely employed in signal processing, energy supply, display technology, solar cells, photodetectors, optical sensors, and medical imaging, which lead to the engineering of optoelectronic devices in both the economic and technological sectors (Colak, 1993; Benkabou et al., 2000; Tamargo, 2002; Adachi, 2009; Deng et al., 2009; Iqbal M. A. et al., 2022). Their characteristics are also size-dependent, and recently, attention has been diverted to the 2023 Nobel Prize in chemistry, which was awarded for the discovery of quantum dots due to their size-dependent display and photodetection applications (Abdullah et al., 2023). In addition to the size, chemical doping and dopant concentration are also important factors that enable the tuning of the material characteristics for the fabrication of novel low-cost, highly efficient devices. In this regard, photonic materials, particularly group II–VI materials, can be tuned to exhibit highly significant and unique properties needed for manufacturing novel and more efficient optoelectronic devices for commercial purposes (Hile et al., 2022). Such properties can be acquired by changing the compounds’ composition so that they can function favorably at a wide range of wavelength spectra. The direct bandgap of group II–VI semiconductors involving cadmium-substituted zinc selenide (CdZnSe) alloys makes them an attractive candidate for use in fabricating various devices in the optoelectronic, photonic, and photovoltaic industries (Colak, 1993; Benkabou et al., 2000; Tamargo, 2002; Adachi, 2009; Iqbal M. A. et al., 2022; Hile et al., 2022; Wang et al., 2022; Abdullah et al., 2023).

The ternary alloys of II–VI group elements are notable semiconductors because their electronic bandgap can be regulated by controlling the dopant content and they are more stable under ambient and extreme conditions (de Melo, 2023; Kurban et al., 2023). These materials exhibit promising potential in connection with photoconductive, photoluminescent, and display devices owing to their flexible bandgap engineering based on the adjustment of dopant concentrations (Burger and Roth, 1984; Razykov, 1988; Nasibov et al., 1989; Sutrave et al., 2000). Transition metals have been frequently used as dopants in these semiconductors, and the alloys of manganese, copper, chromium, nickel, and cadmium are reported in the literature (Nguyen et al., 2022; Jafarova, 2023; Mondal et al., 2023; Sheokand et al., 2023). Various synthetic methodologies, such as vacuum evaporation, chemical bath deposition, the SILAR method, hydrothermal, electro-deposition, and molecular beam epitaxy, have been employed in synthesizing such compounds to explore their potential in fabricating devices with desired outcomes. In particular, CdZnSe semiconductors have been comprehensively synthesized (Schreder et al., 2000; Sutrave et al., 2000; Kishore et al., 2005; Murali and Austine, 2009) to investigate their dielectric, structural, optical, photoluminescence, and magnetic characteristics. In addition, their structural characteristics have also been discussed in the literature (Loglio et al., 2008; Deo et al., 2014), along with their magnetic, luminescence, and dielectric characteristics (Gupta et al., 1995; Margapoti et al., 2012). Despite the availability of multiple reports on the experimental and theoretical studies of noble metals (such as silver and copper) along with transition metal-doped ZnSe semiconductors, there is still a need to explore them further. Along this line, it is necessary to test the suitability of the aforementioned heavily doped semiconductors in selected phases for optimizing dopant content (Xu et al., 2016). Loghina et al. (2019) synthesized quantum dots of CdZnSe alloys and reported their electronic bandgap energy as 2.27 eV. Deng et al. (2009) reported the synthesis of Zn_x_Cd_1-x_Se (0 ≤ X ≤ 1) quantum dots with tunable blue photoluminescence. Their XRD results revealed that the Zn/Cd ratio agrees with Vegard’s law (Vegard, 1921). Recently, Liu et al. (2022) explored CdZnSe quantum dots as the composition of visible displays in blue, red, and green light-emitting diodes. Chen et al. (2022) investigated the impact of the Zn/Cd ratio and studied the photoelectric properties of ternary CdZnSe alloys at Cd contents of 0.1, 0.5, and 0.9. In addition to the experiments, low-cost and effective first-principle approaches have been potentially used to specify a number of physical parameters at ambient pressure in relation to the material’s electrical, optical, thermodynamic, and structural characteristics (Jafarova, 2023; Korozlu et al., 2011; Aarifeen and Afaq, 2017; ul Aarifeen and Afaq, 2019; Ameri et al., 2012; Iqbal et al., 2022b). The optical and electronic properties of ternary Cd_0.25_Zn_0.75_Se semiconductors have been investigated theoretically using the CASTEP code (Korozlu et al., 2011), while thermodynamic properties have been explored in the temperature range of 0–1000 K and a pressure range of 0–10 GPa, using Quantum ESPRESSO software (Aarifeen and Afaq, 2017). Najam et al. explored these ternary semiconductors (Zn_1-x_Cd_x_Se; 0.25 ≤ X ≤ 0.75) to clarify their thermodynamical properties, using the FP-LAPW + lo method within the generalized gradient approximation (GGA) function along with the Hubbard parameter. Thermal conductivity, thermal expansion, internal energy, entropy, Debye temperature, and heat capacities were all calculated (ul Aarifeen and Afaq, 2019). In our recent work, we explored the physical attributes of the Cd_0.25_Zn_0.75_Se alloy under high pressure and discussed its potential for photovoltaic applications (Iqbal et al., 2022b).

From the above discussion, it is evident that CdZnSe wide-bandgap semiconductors are still gaining the attention of the scientific community, although they have been extensively investigated. In this regard, tuning their electronic and optical responses by calibrating material size and the dopant concentration-controlled specific phase is particularly important in fabricating optoelectronic and photovoltaic devices with enhanced stability and applicability in visible displays (Korotcenkov, 2023). The selection of an appropriate dopant and its concentration can lead to novel electronic interactions, such as electron–photon, electron–phonon, and electron–electron interactions, which can be used to induce plasmonics effects (Iqbal et al., 2021a) and attain the tuned optical responses. Keeping this in mind, the present study is designed to modify and improve existing outcomes on CdZnSe alloys by combining the electrical and optical parameters of these semiconducting materials using an all-electronic approach. For this, the optoelectronic and structural properties of Zn_1-x_Cd_x_Se (0 ≤ X ≤ 1) alloys were investigated by performing DFT calculations, including GGA, Engel–Vosko GGA (EV-GGA) functionals, and mBJ potential. A detailed comparison with existing theoretical and experimental studies (Korozlu et al., 2011; Ameri et al., 2012; Loghina et al., 2019; Iqbal et al., 2022b) was also carried out to identify the potential of these alloys in fabricating electronic, photovoltaic, and optoelectronic devices. We examined the cubic symmetry of these materials at varying Cd concentration, with a special focus on the Cd content of x = 0.50, which has never been reported in the cubic phase before, along with the impact of high pressure on the electronic characteristics. This study also aims to encourage experimentalists to analyze our subsequent findings on the alloys for their commercial applications as optoelectronic and photovoltaic devices robust to altitude variations.

## 2 Theoretical method

Kohn–Sham equations were solved via the full-potential linearized augmented plane-wave (FP-LAPW) method (Kohn and Sham, 1965) using the Wien2k code (Blaha et al., 2001) within the DFT framework (Iqbal et al., 2021b). In these evaluations, the interstitial regions and muffin-tin spheres were treated using the FP-LAPW approach to derive eigenvalues together with estimating the physical attributes. The Fourier series was used to understand the basic functions of the method. However, the Schrödinger wave equation was employed to attain the related analytical understanding for the spherical component of the potential. The potential attributed toward the charges was indicated by the spherical harmonics of muffin-tin, which range up to the value of L_max_ = 10, while the product of R_MT_×K_max_ was set at 7 and the G_max_ value was set as 12. The radii for structural generations of muffin-tin spheres were varied for Zn, Cd, and Se, that is, by setting the radii at 2.29, 2.33, and 2.37 a. u. for Zn and Cd and at 2.18, 2.22, and 2.26 for Se for Cd contents of 0.25 ≤ X ≤ 0.75. The energy that separated the core and valence states was set at 10^–6^ Ry, while the irreducible Brillouin zone, which is comparable to the Monkhorst–Pack, has a mesh framework of 12×12×12 k-points (Monkhorst and Pack, 1976). The total energy, force, and charge of the system were optimized by self‐consistent field (SCF) calculations and fixed as 0.00001 Ry, 1 arb. units, and 0.00001 e, respectively. Moreover, for structural optimization, GGA (PBE Sol) (De Waele et al., 2016) was used as an exchange-correlation functional, while EV-GGA (Engel and Vosko, 1993) was used to compute the electronic band structures along with the Tran–Blaha mBJ potential (Singh, 2010). The tuned optical spectra were explored within the mBJ approximation only and provided highly efficient results, comparable to the experimental findings.

## 3 Results and discussion

### 3.1 Structural characteristics

**FIGURE 1:**
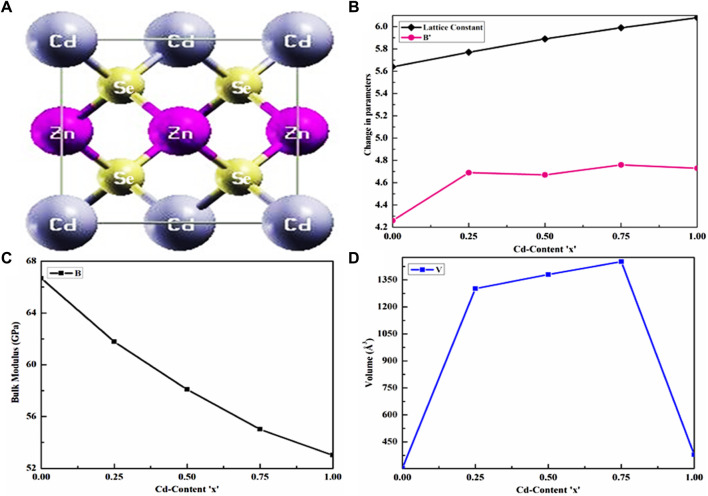
Cd content-dependent structural parameters. **(A)** Cubic structure of Cd_0.50_Zn_0.50_Se alloy. Change in the **(B)** lattice constant and derivative of bulk modulus, **(C)** bulk modulus, and **(D)** ground-state volume.

To explore the exotic characteristics of the crystal at an equilibrium state, the lattice parameters were optimized with a minimum correlating ground-state energy, which was computed by employing Murnaghan’s equation of state (Tyuterev and Vast, 2006), and the findings are summarized in Table 1. The structure of the generated supercell and the variation in structural parameters as a function of the Cd content are shown in Figure 1. To study the structural characteristics and optimize the parameters, a unit cell of CdZnSe was generated. Then, we further investigated the variation of electronic and optical properties as a function of the Cd compositional factor (x), along with the cubic symmetry of these alloys at all Cd concentrations ranging from 0 ≤ X ≤ 1.

**TABLE 1:** Comparison of the lattice constant and bulk modulus computed within GGA with the available literature studies.

Zn_1-x_Cd_x_Se (0 ≤ X ≤ 1)	Lattice constant (Å)			Bulk modulus (GPa)	
	Present work	Experimental	Theoretical	Present work	Literature
0	5.64	5.66 (Caicedo et al., 2000)	5.69 (Korozlu et al., 2011)	66.67	67.05 (Korozlu et al., 2011)
			5.62 (Khenata et al., 2006)		71.82 (Khenata et al., 2006)
			5.67 (Yu et al., 2009)		64.70 (Yu et al., 2009)
0.25	5.77	—	5.79 (Korozlu et al., 2011)	61.77	63.77 (Korozlu et al., 2011)
			5.70 (Bouamama et al., 2009)		
			5.68 (Bouamama et al., 2009)		
0.50	5.89	—	5.95 (Korozlu et al., 2011)	58.09	61.70 (Korozlu et al., 2011)
			5.80 (Bouamama et al., 2009)		
			5.82 (Bouamama et al., 2009)		
0.75	5.99	—	5.99 (Korozlu et al., 2011)	55.01	58.40 (Korozlu et al., 2011)
			5.93 (Bouamama et al., 2009)		
			5.91 (Bouamama et al., 2009)		
1.00	6.08	6.08 (Jamble et al., 2017)	6.09 (Korozlu et al., 2011)	53.01	55.91 (Korozlu et al., 2011)
			6.05 (Deligoz et al., 2006)		65.12 (Deligoz et al., 2006)
			6.06 (Benkhettou et al., 2004)		59.20 (Benkhettou et al., 2004)

Figure 1A shows the CdZnSe cubic crystal structure at a Cd concentration of x = 0.50, while the linear variation in the lattice constant with an increase in the Cd content is presented in Figure 1B. The outcome observed in Figure 1B almost follows Vegard’s law, which states that the lattice constant of an alloy’s crystal structure has a linear relationship with its elemental concentration (Vegard, 1921). The linear increase in both the lattice constant and volume is related to the high Cd concentration, whose atoms substitute Zn sites in the CdZnSe structure. Figures 1C, D show the bulk modulus and volume variation, respectively, with an increase in Cd content. Table 1 summarizes the computed parameters for Cd concentration, including the lattice constant and bulk modulus of the material, as well as their comparison with the existing literature regarding both theoretical and experimental outcomes. The comparison of structural parameters reveals the cubic phase of the investigated alloys at all Cd concentrations, including x = 0.50, which is a unique and important improvement in the existing literature. Recently, a similar cubic symmetry has been reported in the literature for CdZnS semiconductors both theoretically (Iqbal et al., 2022c) and experimentally (Iranmanesh et al., 2017), and it agrees well with the findings of this study. It is also observed that the bulk modulus of CdZnSe semiconductors decreases with an increase in Cd content because the toughness of the material decreases, which indicates its ductile nature. However, ZnSe has a larger bulk modulus compared to its counterparts (CdZnSe alloys), while it has lesser compressibility in comparison with CdSe semiconductors.

### 3.2 Electronic characteristics

**FIGURE 2:**
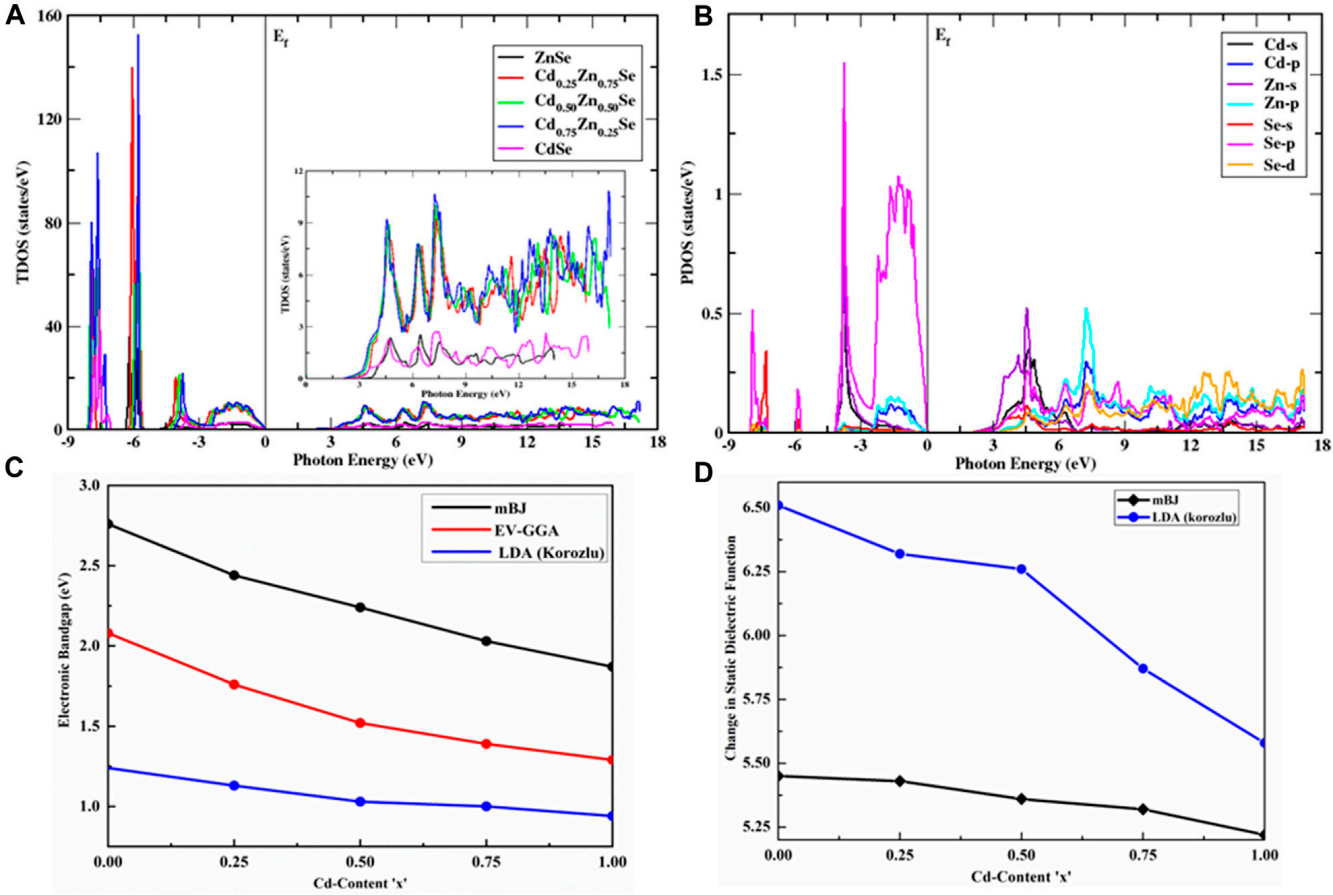
Density of state analysis along with electronic and optical parameters. **(A)** TDOS of Cd_x_Zn_1-x_Se alloys for 0 ≤ *x* ≤ 1. The inset is the zoom of the TDOS within the conduction band and clearly shows its variation with different Cd content. Similarity in their trends confirms cubic symmetry at all the compositional values of Cd, particularly at x = 0.50. **(B)** Illustration of the orbital contribution of Zn, Cd, and Se atoms in Cd_x_Zn_1-x_Se alloy to PDOS at a Cd composition of 75%. **(C)** Dependency of the electronic bandgap energy variation on Cd content. **(D)** Change in $\varepsilon_{1}\left(0\right)$ depending on Cd content along with the literature (Korozlu et al., 2011).

The density of states (DOS) and electronic characteristics of the CdZnSe alloy were investigated within the first Brillouin zone, including their potential applicability in semiconducting electronic devices. The total density of states (TDOS) was investigated to clarify the origin of electronic band structures, along with the exploration of partial DOS (PDOS) to illustrate the orbital contribution of each atom. The electronic band structures show a direct bandgap behavior, where the gap decreases as the concentration of Cd increases. This behavior is well justified with the TDOS being plotted at various Cd concentrations (0 ≤ X ≤ 1), which was computed within the mBJ potential only (as its computational results are very close to the experimental ones) by setting the Fermi energy (E_f_) at 0. DOS spectra show that the total and partial densities of the states of CdZnSe alloys are quite similar to one another, with a minor difference in the intensity, height, and energy of the peak (Figure 2).

The density of states is prominent within the three regions of the valence band, which have multiple peaks. With an increase in Cd content, the intensity increases, and the maximum intensity corresponds to the Cd_0.75_Zn_0.25_Se alloy for both the valence and conduction bands, as can be seen from the blue curve in Figure 2A. The first region in the valence band of DOS is mainly composed of the hybridization of Se-p and Cd-p states, with a minor addition of Se-s and Zn-p states. The second region, where the valence band peak increases with an energy range of -5.92 eV to -6.10 eV, is largely composed of Zn-d, Se-p, and Cd-s states. However, for the third region in the valence band, the peak appears approximately -7.10 eV of incoming light and is composed of Cd-p, Se-s, Se-p, and Zn-p states. It can be concluded that the Se-s and Se-p orbital states contribute to Cd-p and s orbital states, along with Zn-d and Zn-p orbital states that the electrons occupy in CdZnSe alloys (Figure 2B). On the other hand, the conduction band is mainly composed of Zn-p, Zn-s, Se-p, and Se-d states, as well as the contributions of Cd-p and Cd-s orbital states. The zoom of the TDOS within the conduction band is shown in the inset of Figure 2A, where it displays the variation for Cd-based ZnSe alloys, from which one can notice the variation pattern of the band structures and its bandgap dependence. From the similar trend of the DOS pattern, we can confirm cubic symmetry at all compositional values, particularly for x = 0.50.

**FIGURE 3:**
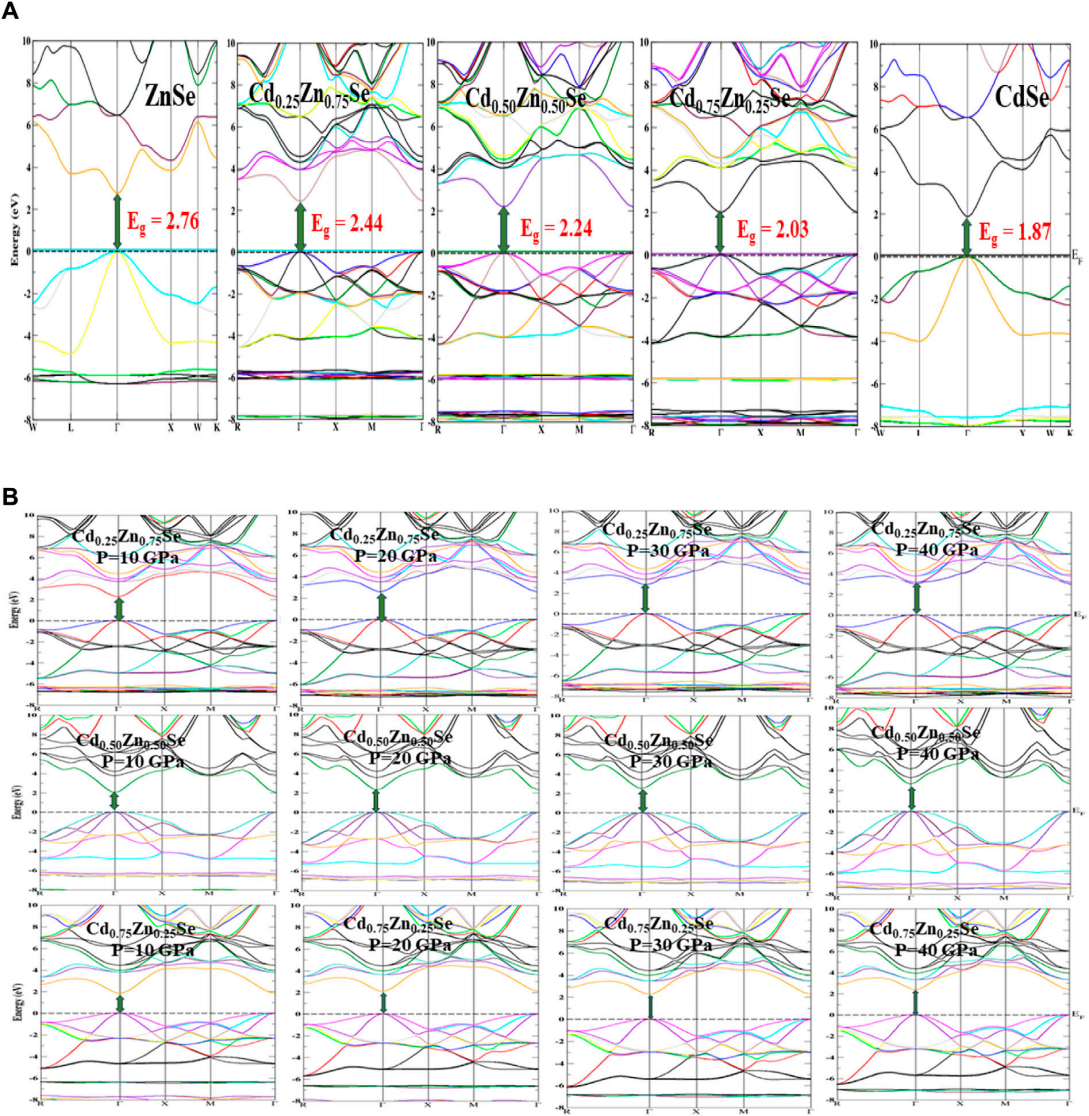
Electronic band structures. **(A)** At varying Cd concentrations (0 ≤ *x* ≤ 1), without considering the pressure impact within the mBJ potential. **(B)** Under the impact of pressure ranging from 10–40 GPa within the EV-GGA functional.

The electronic band structures were generated in the energy range of -8 eV–10 eV for all Cd concentrations (Figure 3A). A close view of the conduction band DOS near the Fermi energy to acknowledge the change in the bandgap with Cd content is presented in Figure 2A (right panel), which clearly shows a varying trend in the TDOS with the increase in the value of Cd concentration. Table 2 summarizes the electronic bandgap (E_g_) of the present research at all Cd contents (0, 0.25, 0.50, 0.75, and 1) with a comparison of previously reported theoretical and experimental studies (Benosman et al., 2000; Hernández, 2002; Benkhettou et al., 2004; Deligoz et al., 2006; Korozlu et al., 2011; Ameri et al., 2012; Jamble et al., 2017; Iqbal et al., 2022b). Based on this table, we confirmed that the present study provides the best calculation to investigate the electronic properties of semiconductor alloys, while the band energies are estimated by EV-GGA and mBJ approximations. mBJ has been proven to have more promising results than EV-GGA because it shifts the conduction band more. Moreover, the direct bandgap nature of the semiconductors has been depicted by the maxima and minima positions of the valence and conduction bands, which are aligned with the same k-space region. The direct bandgap energy values of the materials range from 2.76 eV to 1.87 eV, as displayed in Figure 2C, presenting the semiconductor characteristics, while a comparison of EV-GGA, mBJ, and LDA (Korozlu et al., 2011) data is made in this figure by multi-colored curves. The computed results are in good agreement with the experimental findings (Loghina et al., 2019).

**TABLE 2:** Comparison of bandgap energy and static dielectric constant with the literature studies.

Semiconductor	Electronic bandgap (eV)			Static dielectric constant	
	mBJ	EV-GGA	Literature	mBJ	Literature
ZnSe	2.76	2.08	1.24 (Korozlu et al., 2011)	5.45	6.51 (Korozlu et al., 2011)
			1.18 (Ameri et al., 2012)		
			2.68 (Benosman et al., 2000)		
			2.69 (Hernández,, 2002)		
Cd_0.25_Zn_0.75_ **Se**	2.44	1.76	1.13 (Korozlu et al., 2011)	5.43	6.32 (Korozlu et al., 2011)
			0.83 (Ameri et al., 2012)		
Cd_0.50_Zn_0.50_Se	2.24	1.52	1.03 (Korozlu et al., 2011)	5.36	6.26 (Korozlu et al., 2011)
			0.62 (Ameri et al., 2012)		
**Cd**_**0.75**_ **Zn**_**0.25**_ **Se**	2.03	1.39	1.00 (Korozlu et al., 2011)	5.32	5.87 (Korozlu et al., 2011)
			0.48 (Ameri et al., 2012)		
CdSe	1.87	1.29	0.94 (Korozlu et al., 2011)	5.22	5.58 (Korozlu et al., 2011)
			0.42 (Ameri et al., 2012)		
			1.66 (Benosman et al., 2000)		
			1.90 (Jamble et al., 2017)		

To further investigate the nature of band structures under the influence of high pressure (0–40 GPa), we employed EV-GGA exchange and correlation functional and generated pressure-dependent electronic structures of CdZnSe alloys (Figure 3B). From this analysis, we observed that the pressure significantly shifted the electronic orbitals, and an increase in the electronic bandgap has been noticed. It is also noted that E_g_ varies directly with pressure and that the direct bandgap nature of all these alloys has been retained, suggesting the use of these wide-bandgap alloys as optical sensors that can function at varying altitudes.

### 3.3 Optical properties

The mechanism of light–matter interactions is of great significance in studying the photonic and optoelectronic properties of a material. This mechanism provides a better understanding of the electron–photon interaction and the process of energy emission by hot electrons during cooling, which can be used to investigate the optical response of the material. The excitation spectra hold important information and are observed due to the shifts in the electron from an occupied to an unoccupied state, which are related to the system’s linear response and joint densities. The linear optical response of a material is explained by the behavior of the complex dielectric function, which can mathematically be expressed as follows (Fox, 2001):

$$\varepsilon \left(\omega \right)=\varepsilon_{1}\left(\omega \right)+i\varepsilon_{2}\left(\omega \right)$$ (1)

Herein, $\varepsilon_{1}\left(\omega \right)$ and $\varepsilon_{2}\left(\omega \right)$ present the real and imaginary portions of the dielectric function, respectively. The real portion depicts the material’s polarization and also indicates the dielectric function at an irradiation frequency of 0 Hz. The imaginary part helps to determine the absorption. All the optical parameters can be approximated from the analyses of the complex dielectric function. The static dielectric constant ( $\varepsilon_{1}\left(0\right)$ ) is also an important parameter since it is closely related to the optical bandgap of the material; that is, the optical bandgap can be computed using the relation *ε*_1_ (0) ≈ 1 + (*ħω*/*E*_*g*_)^2^. The Kramers–Kronig relationship can be used to relate and compute both the real and imaginary portions of the dielectric function as follows (Johnson, 1975):

$$\varepsilon_{1}\left(\omega \right)=1+\frac{2}{\pi }\int_{0}^{\infty }\frac{\omega^{/}\varepsilon_{2}\left(\omega \right)}{\omega^{/2}-\omega^{2}}d\omega^{/}$$ (2)

$$\varepsilon_{2}\left(\omega \right)=-\frac{2\omega }{\pi }P\int_{0}^{\infty }\frac{\left(\varepsilon_{1}\left(\omega^{/}\right)\left.-1\right)d\omega \right.}{\omega^{/2}-\omega^{2}}$$ (3)

where P represents the momentum matrix and ω ^/^ represents the collective density of states.

The dielectric function can also be used to calculate other optical quantities, such as refractive index $n\left(\omega \right)$ and extinction coefficient $k\left(\omega \right)$ , which provide information about the decrease in the speed of radiation when it passes through a medium and absorption, respectively (Fox, 2001). Both $n\left(\omega \right)$ and $k\left(\omega \right)$ can mathematically be expressed in the forms as follows:

$$n\left(\omega \right)=\frac{1}{\sqrt{2}}{\left[\sqrt{\left\{{\varepsilon_{1}}^{2}\left(\omega \right)+{\varepsilon_{2}}^{2}\left(\omega \right)\right\}}+\varepsilon_{1}\left(\omega \right)\right]}^{1/2}$$ (4)

$$k\left(\omega \right)=\frac{1}{\sqrt{2}}{\left[\sqrt{\left\{{\varepsilon_{1}}^{2}\left(\omega \right)+{\varepsilon_{2}}^{2}\left(\omega \right)\right\}}-\varepsilon_{1}\left(\omega \right)\right]}^{1/2}$$ (5)

In addition, to calculate the optical absorption of the material, the absorption coefficient can be used by employing the imaginary component of the material’s refractive index (Fox, 2001). This coefficient also provides useful information about the decay of radiation with an increase in distance. Mathematically, it can be presented as follows:

$$\alpha \left(\omega \right)=\frac{4\pi k\left(\omega \right)}{\lambda_{0}}=\frac{\omega }{nc}\varepsilon_{2}\left(\omega \right)$$ (6)

Reflection spectra that are used to investigate the response of incident light to the material’s surface can be written as follows (Fox, 2001):

$$R\left(\omega \right)=\frac{{\left(n\left(\omega \right)-1\right)}^{2}+k^{2}\left(\omega \right)}{{\left(n\left(\omega \right)+1\right)}^{2}+k^{2}\left(\omega \right)}$$ (7)

The electrical transport to high frequencies is referred to as optical conductivity, which is a contact-free measurement and sensitive to charge responses. Its real part can be determined from the imaginary part of the dielectric coefficient such that (Fox, 2001):

$$Re\sigma \left(\omega \right)=\frac{\omega .\varepsilon_{2}}{4\pi }$$ (8)

The interacting electrons’ energy loss function $L\left(\omega \right)$ can be expressed as a function of the dielectric function as follows (Fox, 2001):

$$L\left(\omega \right)=Im\left(-\frac{1}{\varepsilon \left(\omega \right)}\right)=\frac{\varepsilon_{2}}{\left(\varepsilon_{1}^{2}\left(\omega \right)+\varepsilon_{2}^{2}\left(\omega \right)\right)}$$ (9)

**FIGURE 4:**
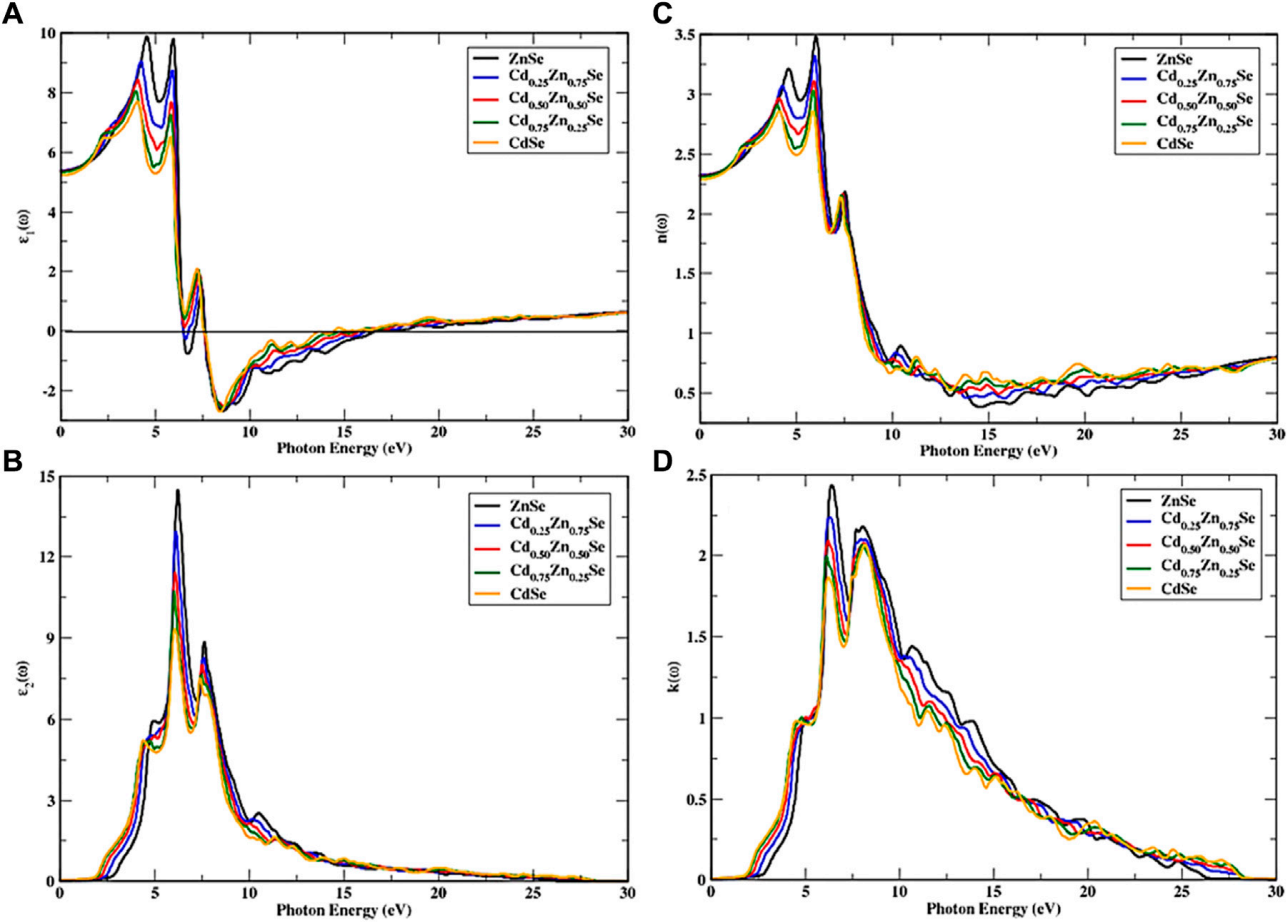
Approximated dielectric function and optical parameters as a function of incident photon energy. **(A)** $\varepsilon_{1}\left(\omega \right)$ , **(B)** $\varepsilon_{2}\left(\omega \right)$ , **(C)** $n\left(\omega \right)$ , and **(D)** $k\left(\omega \right)$ variations with Cd concentrations (0 ≤ *x* ≤ 1).

**FIGURE 5:**
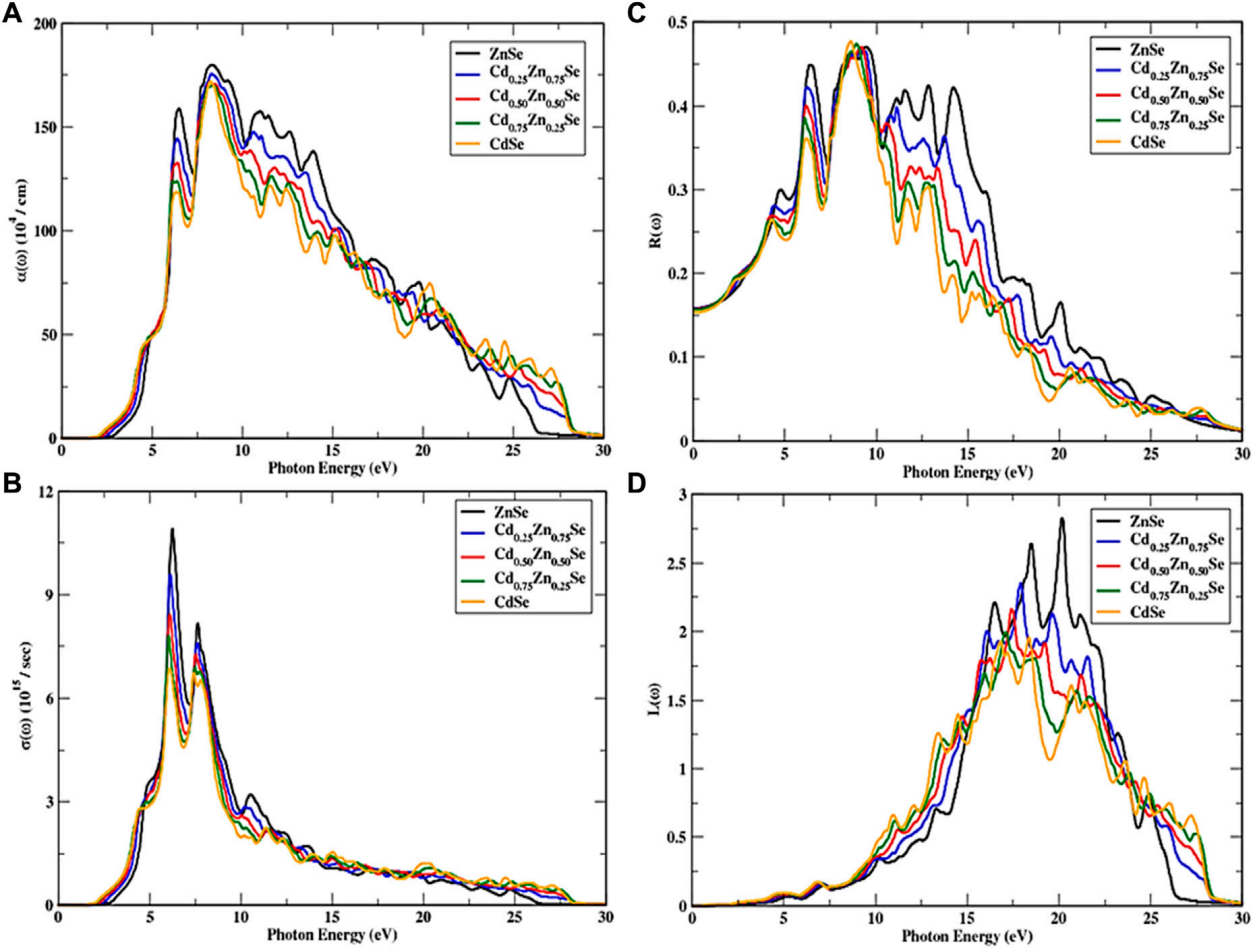
Variations in optical parameters over the incident photon energy depending on the Cd concentration (0 ≤ *x* ≤ 1). **(A)** Absorption, **(B)** Optical conductance, **(C)** Reflectance, and **(D)** Energy loss function.

CdZnSe alloys show an isotropic optical response, owing to their cubic symmetry. To further study the optical properties of the alloys, a dense framework of k points was used to investigate the incident radiations of approximately 30 eV by using the mBJ potential. Figures 4, 5 show the spectra of all optical parameters.

Figure 4A shows a fluctuating trend in the peak position, which is observed to be rapidly increased in the first interval and then showed a minor drop, which again turned into an increase with the maximum peak position for the real part of the dielectric function and seems to have more transitions ranging from 2.16 eV to 5.91 eV. With high Cd concentrations, the highest peak tends to decline and shifts toward lower incident energy. For the incident photons ranging from 6.41 eV to 16.53 eV, there exists a maximum reflectance; hence, they show a metallic nature below zero-unit values, which is applicable in shielding the vacuum and intense UV radiation. After the energy range of 17.93 eV, the real part again became positive, and a static spectrum was observed, indicating that these materials do not interact with incoming radiations and, hence, can be used in optical shields for this particular energy range. By increasing the Cd concentration in alloys, the value of the static dielectric constant decreases from a maximum value that corresponds to pure ZnSe; that is, it decreases as a function of Cd content from 5.45 to 5.22, and a minimum value corresponds to CdSe (Figure 2D). The results for Cd_0.25_Zn_0.75_Se alloys agree well with the existing literature (Iqbal et al., 2022b). With the increase in Cd concentration, the threshold energy of the imaginary part of the dielectric constant keeps on decreasing, inducing a decrease in the bandgap, as observed from the band structures. This causes the direct interband transitions at these points from valence to conduction bands, while out of these points, the imaginary part of the dielectric coefficient is found to increase at the incoming radiation energy of approximately 5.37 eV, where the first peak appears. Figure 4B shows the four major peaks observed in the range of radiation energy from the threshold level to 10.59 eV, wherein the highest threshold corresponds to ZnSe; the highest major peak which is different depending on the Cd concentration experiences a drop from 14.55 to 9.31 units with the increase in the concentration of Cd, whereas the spectrum for CdSe exhibits lower energies. On the other hand, these semiconductors have a low absorption coefficient at and above 22.48 eV because the energy spectra do not occur prominently and disappear at 28.20 eV and higher.

The investigated CdZnSe material has a cubic symmetry; hence, the refractive index is similar in both transverse directions, wherein the static refractive index of the alloy experiences a decline with an increase in Cd concentration and the main active peak region of incoming energy ranges from 2.16 to 7.41 eV (Figure 4C). Furthermore, new peaks appear depending on Cd content, and these diverse peaks last at 27.50 eV of incoming radiation. The overall pattern remains the same except for the decrease in spectrum intensity with Cd content. The refractive index at the irradiation frequency of zero (*n*(0)) shows a variation with the Cd concentration in the range of 2.32 to 2.28 units, and a steady response of the material can be seen at the incident radiation energy of approximately 28 eV and above. Figure 4D shows the variation in extinction coefficient and the drop in peak height from 2.43 to 1.87 units along with the decrease in threshold energy from 2.70 to 1.67 eV with the variation of Cd concentrations (0 ≤ X ≤ 1). A decreasing trend in $n\left(\omega \right)$ and $k\left(\omega \right)$ is observed with an increase in Cd concentration, and there is no spectrum present above the incident photons’ energy range of 28.16 eV, implying that the absorption at these high energies is ignorable (see Figures 4B, D).

Figure 5A shows the optical absorption spectra of the materials, which can be attributed to the imaginary part of the dielectric function. The absorption peak of CdZnSe semiconductors decreases from 180.65 to 163.49 units, whereas ZnSe exhibits the extreme peak, while the absorption coefficient of CdZnSe (at x = 0.25) is 175.96 arb. units at 8.29 eV, which is higher than that of its counterparts (at x = 0.50, 0.75, and 1). A steady pattern of the spectrum is observed above the incident photon energy of 28.39 eV. There is no absorption if the energy of incident photons is below the bandgap energy, while maximum absorption takes place in the range of 5.99 eV–21.26 eV, along with the newfangled peaks that occurred due to the valence to conduction band electronic transitions. The overall decreasing trend in absorption along with the increase in Cd content is noted, which is in accordance with the literature (Iqbal et al., 2022b). The change in the optical conductivity of CdZnSe semiconductors is shown in Figure 5B, and from this, it can be seen that the conductance value remains 0 if the photon energy is less than the bandgap energy of the material. Above the bandgap energy of the incident radiation, it starts to sharply increase up to its maximum value, while the peak height decreases to 6.87 from 10.91 units with an increase in the Cd concentration. Moreover, in the energy range of 4.32 eV–13.16 eV, the spectra exhibit a greater number of peaks. A steady response is also observed at a high incident photon energy of 28.12 eV. The ZnSe semiconductor has the maximum optical conductivity value at an incident energy of 6.04 eV, while this material presents a high conductivity in the range of incident photon energy of 6.04 eV–7.79 eV, which proved it to be optically active in this region. In general, all these semiconductors are optically active and have a high conductivity in the energy range of 4.37 eV–10.82 eV.

When the incident photon energy increases, an increase in reflectance spectra is observed, as shown in Figure 5C. A peak shift is also observed in conjunction with the wider spectra; that is, the peak moves toward the lower energies as the Cd concentration (0 ≤ X ≤ 1) increases, while its height decreases, and the humps in the peak are observed in the energy range of 4.15 eV–27.50 eV. Although CdSe shows the maximum reflectance at a certain photon energy, the reflectance of the alloys drops with an increase in Cd concentration from 0 to 1 at most photon energies. The reflectance values at the irradiation frequency of 0 decreased from 0.161 to 0.154 when subjected to Cd concentrations, and new peaks have been observed in the incident photon energy of 4.15 eV–19.99 eV. Peak height is observed to have increased and the main peak that appears at 9.43 eV for ZnSe shifted further up. The minimum reflectance was observed for the CdSe semiconductor from and above the incident energy of 10 eV, while ZnSe shows a reverse trend in this region. The main reflectance region lies in the incoming radiation range of 5.63 eV–16.93 eV, with dispersed peaks. However, if the incident electron energy is less than the material’s bandgap energy, then there is no loss of electron energy, showing no observation of electron scattering at all. Inelastic electron scattering can be observed at specific energy levels that are higher than bandgap energy, which forms a spectrum that is proportional to the incoming radiation. Figure 5D shows an observation of higher electron energy loss for ZnSe semiconductors at a high energy value of 20.13 eV, with a value of 2.82 arb. units, while it decreases with an increase in Cd concentrations. While the main peaks are detected in the range of 10.12 eV–27 eV for photon energy, the peak heights shift to low energy values with an increase in Cd concentrations. However, the electron energy loss function is ignorable when the incident photon energy is above 28 eV, and it can also be neglected if the incident photon energy is under 3.87 eV with a peak detection range of 10.12 eV–27 eV.

## 4 Conclusion

We investigated the structural and optoelectronic characteristics of CdZnSe semiconductors with varying Cd concentration by utilizing the FP-LAPW method within DFT. Our outcome proves that the alloys managed to have a cubic symmetry, while the lattice constant increases linearly as the Cd concentration increases. However, their bulk modulus exhibits a reverse trend with the augmentation of Cd concentration. The semiconducting properties of the materials are predicted from the band structure and density of state analysis, whereas the bandgap is observed to decrease with an increase in Cd content. However, under the influence of pressure, a reverse trend in electronic bandgap is observed. These alloys exhibit isotropic optical properties that are closely related to their cubic symmetry, while the optical spectrum is favorable for visible display. Hence, they can potentially be applied in the field of optoelectronics, such as photodetectors and optical lenses in the industrial sector of photonics and photovoltaics.

## Data Availability

The original contributions presented in the study are included in the article/Supplementary Material; further inquiries can be directed to the corresponding authors.
